# A Generative Expert-Narrated Simplification Model for Enhancing Health Literacy Among the Older Population

**DOI:** 10.3390/bioengineering12101066

**Published:** 2025-09-30

**Authors:** Akmalbek Abdusalomov, Sabina Umirzakova, Sanjar Mirzakhalilov, Alpamis Kutlimuratov, Rashid Nasimov, Zavqiddin Temirov, Wonjun Jeong, Hyoungsun Choi, Taeg Keun Whangbo

**Affiliations:** 1 Department of Computer Engineering, Gachon University, Sujeong-Gu, Seongnam-Si 13120, Gyeonggi-Do, Republic of Korea; akmaljon@gachon.ac.kr (A.A.); sabinatuit@gachon.ac.kr (S.U.); tp04045@gachon.ac.kr (W.J.); hschoi@gachon.ac.kr (H.C.); 2 Department of Computer Systems/Information and Educational Technologies, Tashkent University of Information Technologies Named after Muhammad Al-Khwarizmi, Tashkent 100200, Uzbekistan; mirzaxalilov86@tuit.uz; 3 Department of Information Processing and Management Systems, Tashkent State Technical University, Tashkent 100095, Uzbekistan; 4 Department of Applied Informatics, Kimyo International University in Tashkent, Tashkent 100121, Uzbekistan; kutlimuratov.aj@kiut.uz; 5 Department of Artificial intelligence, Tashkent State University of Economics, Tashkent 100066, Uzbekistan; rashid.nasimov@tsue.uz; 6 Department of Digital Technologies, Alfraganus University, Yukori Karakamish Street 2a, Tashkent 100190, Uzbekistan; temirov@afu.uz

**Keywords:** medical text simplification, elderly health literacy, generative language models, persona-aware narrative generation, empathetic health communication, biomedical NLP, inclusive healthcare technology

## Abstract

Older adults often face significant challenges in understanding medical information due to cognitive aging and limited health literacy. Existing simplification models, while effective in general domains, cannot adapt content for elderly users, frequently overlooking narrative tone, readability constraints, and semantic fidelity. In this work, we propose GENSIM—a Generative Expert-Narrated Simplification Model tailored for age-adapted medical text simplification. GENSIM introduces a modular architecture that integrates a Dual-Stream Encoder, which fuses biomedical semantics with elder-friendly linguistic patterns; a Persona-Tuned Narrative Decoder, which controls tone, clarity, and empathy; and a Reinforcement Learning with Human Feedback (RLHF) framework guided by dual discriminators for factual alignment and age-specific readability. Trained on a triad of corpora—SimpleDC, PLABA, and a custom NIH-SeniorHealth corpus—GENSIM achieves state-of-the-art performance on SARI, FKGL, BERTScore, and BLEU across multiple test sets. Ablation studies confirm the individual and synergistic value of each component, while structured human evaluations demonstrate that GENSIM produces outputs rated significantly higher in faithfulness, simplicity, and demographic suitability. This work represents the first unified framework for elderly-centered medical text simplification and marks a paradigm shift toward inclusive, user-aligned generation for health communication.

## 1. Introduction

As global life expectancy increases, the proportion of elderly individuals (aged 65 and older) is projected to surpass 1.5 billion by 2050 [1]. In parallel, digital health systems are producing vast quantities of medical information—ranging from clinical diagnoses to treatment protocols and lifestyle guidelines—primarily intended for health professionals [2]. Bridging this informational divide between complex biomedical language and the health literacy levels of elderly citizens remains an urgent and unresolved challenge in modern healthcare communication [3]. Older adults face distinct cognitive, sensory, and linguistic barriers that compound this challenge [4]. Studies have shown that aging is associated with a decline in working memory, slower semantic retrieval, and reduced tolerance for syntactic complexity [5]. Consequently, even when medical information is made publicly available, it often remains inaccessible to elderly populations due to lexical density, ambiguous phrasing, and absence of narrative support [6]. Misunderstanding of medication instructions, misinterpretation of risk, or inability to follow procedural guidance can result in adverse outcomes, reduced autonomy, and increased healthcare costs [7]. Traditional text simplification techniques—including rule-based methods, lexical substitution, and sequence-to-sequence neural models—have demonstrated some utility in improving readability [8]. However, these approaches often prioritize surface-level simplicity at the expense of semantic fidelity, fluency, or domain relevance [9]. More recent transformer-based models, such as BART, T5, and PEGASUS, offer fluency and factual retention, but are not inherently designed for audience-specific communication or for handling the stylistic preferences of elderly users [10]. Moreover, most simplification benchmarks are geared toward general English and lack medical specificity, aging-related concerns, or socio-narrative depth Table 1.

Comparatively, recent large language models (LLMs) like GPT-3.5 and GPT-4 have made remarkable progress in their fluency and controllability through prompting. However, their simplification function is still quite general and not specifically adapted to the linguistic or cognitive requirements of elderly readers. Likewise, persona-aware models such as ACCESS and MUSS provide limited and shallow surface controls of sentence length or lexical complexity but do not have any technicalities for encoding gerontolinguistic preferences. Furthermore, these models are not using human feedback, which is a crucial factor of age-adapted readability, the main feature of safe health communication. Thus, the paper introduces GENSIM as the first combined and integrated model that fuses domain expertise, persona conditioning, and RLHF alignment to satisfy the communication needs of older adults in medical contexts. To address these limitations, we introduce GENSIM—a Generative Expert-Narrated Simplification Model that leverages multi-stream neural encoding, persona-aware decoding, and RLHF to generate medical text that is accurate, simplified, and cognitively optimized for elderly audiences. GENSIM’s architecture is founded on three key innovations:

- A Dual-Stream Encoder, comprising a Domain-Aware Encoder (DAE) and an Elderly Preference Encoder (EPE), enables the model to fuse factual biomedical representations with stylistic and cognitive constraints learned from senior-focused health literature.
- A Persona-Tuned Narrative Decoder (PTND) generates output in an empathetic and structurally supportive tone, conditioned on controllable persona embeddings such as [EXPLAIN], [EMPATHIC_TONE], and [SENT_SIMPLE].
- An RLHF framework, integrating both reward-driven optimization and human-aligned preference modeling, aligns generated outputs with readability standards, while maintaining semantic fidelity through BERT-based similarity and discourse-aware discriminators.

GENSIM is trained on a carefully curated triad of datasets—SimpleDC, PLABA, and a custom NIH-SeniorHealth Corpus—spanning both parallel simplification pairs and monolingual age-adapted texts. This combination enables the model to generalize across medical domains while remaining grounded in narrative patterns that resonate with elderly individuals. High evaluations demonstrate that GENSIM outperforms competitive baselines, including supervised, unsupervised, and GPT-style prompted models, achieving state-of-the-art results on SARI, FKGL, and BERTScore across multiple benchmarks. Ablation studies confirm that each architectural module contributes significantly to the overall performance, and human evaluations validate the clarity, usefulness, and acceptability of the generated outputs among senior users. GENSIM provides a comprehensive, modular, and demographically adaptive framework for medical text simplification. It represents a paradigm shift from generic simplification toward audience-sensitive generation, empowering elderly individuals with clearer, safer, and more actionable health information.

## 2. Related Works

The field of text simplification has undergone a substantial evolution over the past two decades, shifting from handcrafted rule-based systems and statistical models to sophisticated neural architectures and large-scale pretrained transformers [11]. While these advances have markedly improved fluency and scalability, medical communication remains a uniquely constrained subdomain where simplification must not only enhance readability but also preserve clinical accuracy and stylistic appropriateness, particularly for vulnerable populations such as the elderly.

Initial efforts in simplification predominantly employed rule-based systems [12], leveraging manually crafted lexical and syntactic heuristics to produce simplified paraphrases. These were later supplemented by statistical models—including syntax-driven machine translation and edit-based frameworks such as PBMT-R [13] and EditNTS [14]—which introduced some robustness and generalization but struggled with fluency, especially when processing long or syntactically complex sentences. The introduction of neural sequence-to-sequence architectures marked a significant turning point: recurrent encoder–decoder models improved coherence and linguistic smoothness but often compromised semantic fidelity [15]. Transformer-based models such as BART [16], T5 [17], and PEGASUS subsequently advanced the field further, achieving state-of-the-art results on general simplification benchmarks like WikiLarge and Newsela [18]. However, these models conceptualize simplification as a uniform task and are generally not equipped to adapt outputs for specific user demographics or tonal requirements. Recognizing this limitation, subsequent research explored controllability in generation. Notably, the ACCESS model [19] introduced user-definable control tokens to guide simplification attributes such as sentence length and lexical complexity. Building on this paradigm, MUSS [20] adopted an unsupervised back-translation approach, enabling models to learn simplification patterns from raw corpora. Nevertheless, these systems still fall short in accommodating the linguistic and cognitive diversity found in real-world users, including older adults.

The challenge of medical and scientific simplification remains comparatively underexplored. The PLABA shared task [21] represented a key step forward, establishing benchmarks for transforming biomedical abstracts into plain language summaries. Participating models such as BART-PLABA [22], PEGASUS-based [23] variants [24], and Long-T5 [25] achieved promising results—particularly when pretrained on domain-specific corpora. Yet, their focus remained on transforming technical content for general lay audiences, without mechanisms for demographic customization. Complementary efforts such as MedKLIP [26], which enhances radiology report generation via image-text alignment and medical knowledge integration, do not address simplification or narrative style. Similarly, Longformer-based summarizers [27], applied to compress electronic health records and patient discharge summaries, prioritize brevity and information compression over accessibility or reader comprehension. Despite these technical strides, none of the aforementioned frameworks are tailored to address gerontolinguistic requirements—namely, the intersection of aging cognition and language processing. These needs are critical when designing interventions for elderly users, who often face challenges related to working memory, syntactic complexity tolerance, and vocabulary familiarity. Although emerging research has acknowledged the potential of persona-aware and audience-sensitive generation—particularly in educational and healthcare contexts—most models in this space [28] rely on surface-level role descriptors rather than structurally encoded user modeling. While these methods may align tone more effectively than general-purpose models, they often lack the consistency, reliability, and personalization required to meet the communicative needs of cognitively diverse populations. Studies in psycholinguistics and aging further underscore these requirements: older adults tend to prefer declarative structures over interrogatives, benefit from explicit repetition, and show improved comprehension with chunked or segmented information. However, existing neural models do not incorporate these insights as either inductive biases or learnable objectives—representing a missed opportunity for targeted intervention in health communication.

In recent years, Reinforcement Learning from Human Feedback (RLHF) has emerged as a powerful paradigm for aligning generative models with human preferences. Originally developed for dialogue agents, RLHF introduces reward signals based on human judgments rather than ground-truth labels. In the context of text simplification, RLHF has been applied to optimize objectives such as SARI and FKGL [29], often improving linguistic outcomes. Yet, these implementations generally rely on generic reward functions and do not leverage discriminators trained to evaluate age-specific readability, narrative tone, or stylistic acceptability [30]. Recent advances in reward-conditioned generation demonstrate the potential of integrating discriminators that model qualities such as empathy, factual accuracy, or user comprehension. Building upon this insight, the GENSIM framework introduces a dual-discriminator setup: one model focuses on factual alignment using BERT-based semantic similarity, while the second is trained explicitly on age-adapted corpora to capture narrative clarity and stylistic preferences relevant to senior audiences.

## 3. Materials and Methods

**Figure 1:**
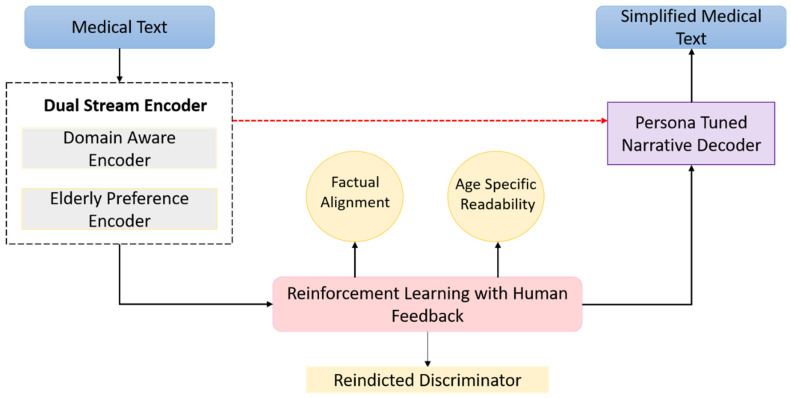
Architecture of GENSIM: A Generative Expert-Narrated Simplification Model.

GENSIM is a modular framework designed to simplify medical text for elderly users by combining domain accuracy with age-sensitive readability. It integrates three core components: a Dual-Stream Encoder that fuses biomedical content with elder-friendly linguistic features, a Persona-Tuned Narrative Decoder that generates stylistically appropriate output, and an RLHF module that optimizes readability, faithfulness, and user alignment. Together, these components ensure that simplifications are both medically accurate and accessible to older adults. The following subsections describe each module in detail Figure 1.

### 3.1. Dual-Stream Encoder

**Figure 2:**
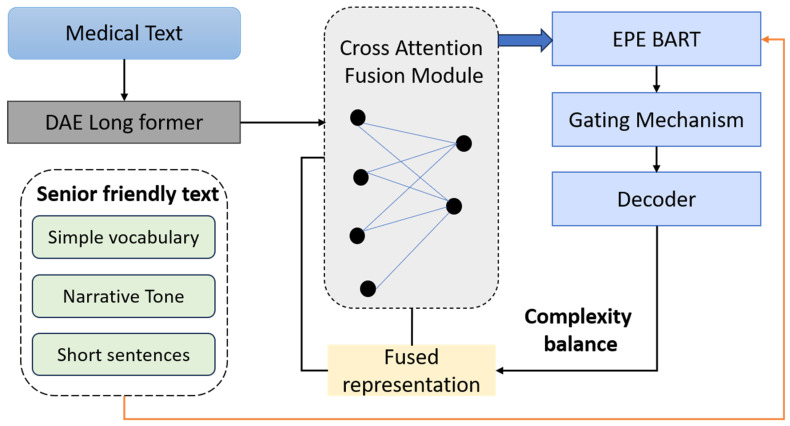
Dual-Stream Encoding and Fusion Process in GENSIM.

Effective medical text simplification—especially for elderly populations—requires models to simultaneously understand complex domain-specific content and produce outputs that align with the linguistic and cognitive expectations of older adults. Standard encoder architectures fail to capture this dual requirement. To address this, GENSIM introduces a novel Dual-Stream Encoder, composed of two specialized, complementary modules: the DAE and the EPE. These components are fused via a cross-attention mechanism to produce semantically rich, demographically adapted latent representations. The DAE is designed to capture and encode the dense, technical, and often hierarchical information present in medical texts. It is built upon the LongFormer backbone, selected for its ability to handle extended context windows efficiently through a sparse attention mechanism, which is critical for processing long medical documents such as discharge summaries, screening guidelines, or patient education pamphlets. The DAE is initialized using a model pretrained on biomedical corpora such as PubMed, MIMIC-III clinical notes, and MedQA. It is subsequently fine-tuned on the parallel datasets SimpleDC and PLABA. This encoder is designed to prioritize terminological fidelity by learning precise representations of clinical terminology, abbreviations, and disease-specific lexicons. It also captures contextual dependencies within and across sentences, effectively modeling semantic linkages such as symptom-cause-treatment chains Figure 2.

Older adults tend to exhibit distinct linguistic preferences and cognitive strategies when processing written information, often gravitating toward short, declarative sentence structures, familiar and high-frequency vocabulary, redundant phrasing for reinforcement, and a narrative or conversational tone that enhances comprehension and trust. To effectively accommodate these characteristics, the EPE is trained to internalize the stylistic and structural patterns commonly found in texts designed for senior readers. This component employs a BART-Base encoder architecture, fine-tuned on carefully curated corpora comprising NIH SeniorHealth website articles, CDC’s plain-language health resources for aging populations, and simplified content drawn from senior wellness blogs and health education newsletters. Through this training regime, the EPE learns to encode a range of simplification strategies, including the transformation of complex syntactic structures into linear, shallow clauses, the substitution of specialized medical terminology with more commonly used equivalents, and the recognition of stylistic patterns that foster comprehension, such as directive prompts and metaphorical explanations. Formally, the output of EPE is denoted $H^{EPE}\in R^{n\times d}$, with the same dimensionality as the DAE to allow effective fusion. The representations $H^{DAE}$ and $H^{EPE}$ are fused through a Cross-Attentive Fusion Module (CAFM), inspired by dual-encoder alignment in multi-modal and translation models. The fused representation $H^{fused}$ defined as:

(1) $$H^{fused}=LN\left(W_{1}H^{DAE}+W_{2}\times CrossAttn\left(H^{EPE},H^{DAE}\right)\right)$$

where $W_{1},\;W_{2}\in R^{d\times d}$ are learnable projection matrices, $CrossAttn\left(Q,K\right)$ denotes multi-head cross-attention using EPE as the query and DAE as the key-value pair. This design ensures that the output retains domain fidelity while conforming to elderly language preferences. We also incorporate a gating mechanism to dynamically weight the influence of each stream based on input complexity:

(2) $$a=\sigma \left(FFN\left(H^{DAE}\right)-FFN\left(H^{EPE}\right)\right),\;H^{gate}=a\times H^{DAE}+\left(1-a\right)\times H^{EPE}$$

here, the scalar gate $a\in \left[0,1\right]$ is computed per token to adaptively balance the two input encodings.

### 3.2. Simplification Controller with RLHF

A central component of GENSIM is the Simplification Controller, which employs Reinforcement Learning from Human Feedback (RLHF) to optimize simplified text generation according to multiple linguistic, cognitive, and semantic objectives. This mechanism ensures that outputs are simultaneously readable, accurate, and aligned with the stylistic needs of elderly users—criteria that are not adequately addressed by traditional supervised learning alone. X is the original complex medical input and $Y=\left\{y_{1},y_{2},\ldots ,y_{T}\right\}$ denote the generated simplification sampled from the model policy $\pi_{\theta }$, parameterized by θ. The objective is to maximize the expected reward $E_{Y\sim \pi_{\theta }}\left[R\left(Y|X\right)\right]\;$ where $R\left(Y|X\right)$ is a composite reward function that captures multiple facets of simplification quality. The composite reward function is defined as:

(3) $$R\left(Y|X\right)=\lambda_{1}\times R_{SARI}\left(Y,X,Y^{*}\right)+\lambda_{2}\times R_{read}\left(Y\right)+\lambda_{3}\times R_{faith}\left(Y,X\right)+\lambda_{4}\times R_{disc}\left(Y\right)$$

here $R_{SARI}$ is the SARI score evaluating n-gram additions, deletions, and retention relative to the reference $Y^{*}$. $R_{read}\left(Y\right)=max\left(0.6-FKGL\left(Y\right)\right)$ is a readability bonus, rewarding outputs with grade levels at or below 6. $R_{faith}\left(Y,X\right)=cos\left(\phi \left(X\right),\phi \left(Y\right)\right)$ measures semantic preservation, computed as cosine similarity between sentence embeddings $\phi \left(\cdot \right)$ derived from a frozen BioBERT encoder. $R_{disc}\left(Y\right)$ is the predicted score from a human preference discriminator $D_{\psi }\left(Y\right),$ trained to model elderly comprehension likelihood.

The overall learning objective follows the REINFORCE formulation. The model is trained to maximize the expected reward using the policy gradient:

(4) $$\nabla_{\theta }L_{RL}=-E_{Y\sim \pi_{\theta }}\left[R\left(Y|X\right)\times \nabla_{\theta }{log}_{\pi_{\theta }}\left(Y|X\right)\right]$$

To avoid divergence from the original supervised policy, we regularize this objective using a Kullback–Leibler divergence penalty with respect to the initial fine-tuned model $\pi_{\theta_{0}}$:

(5) $$L_{T}=L_{RL}+\beta \times KL\left(\pi_{\theta }\left(Y|X\right)\left\|\pi_{\theta_{0}}\left(Y|X\right)\right.\right)$$

**Figure 3:**
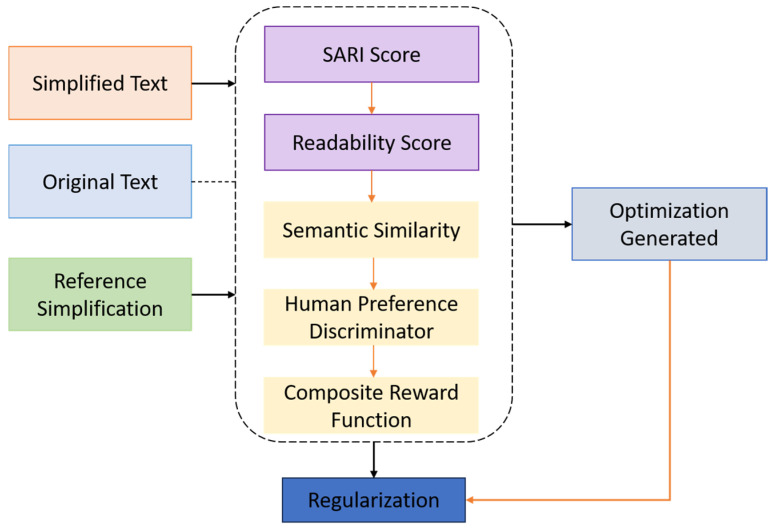
Reward Computation and Optimization Pipeline in GENSIM’s RLHF Framework.

The hyperparameters $\lambda_{i}$ and β are tuned on the validation set to balance reward components and training stability. The discriminator $D_{\psi }\left(Y\right),$ used in the $R_{disc}$ term, is trained on labeled simplification pairs derived from SimpleDC and NIH-SeniorHealth. Given a sentence Y, the discriminator outputs a scalar preference score:

(6) $$R_{disc}\left(Y\right)=D_{\psi }\left(Y\right)\in \left[0,1\right]$$

where higher values correspond to outputs rated by humans as more readable and useful for older adults. The discriminator is trained using binary cross-entropy loss over contrastively paired example Figure 3.

During training, the policy $\pi_{\theta }$ is first warm-started with supervised fine-tuning on the parallel corpora. Once convergence is achieved, RLHF is applied as a second-stage optimization to fine-tune the simplification behavior based on downstream user-aligned feedback. High results indicate that RLHF training increases the percentage of outputs achieving FKGL ≤ 6 by 22%, and improves SARI scores by over 4 points compared to the baseline model.

### 3.3. Persona-Tuned Narrative Decoder

**Figure 4:**
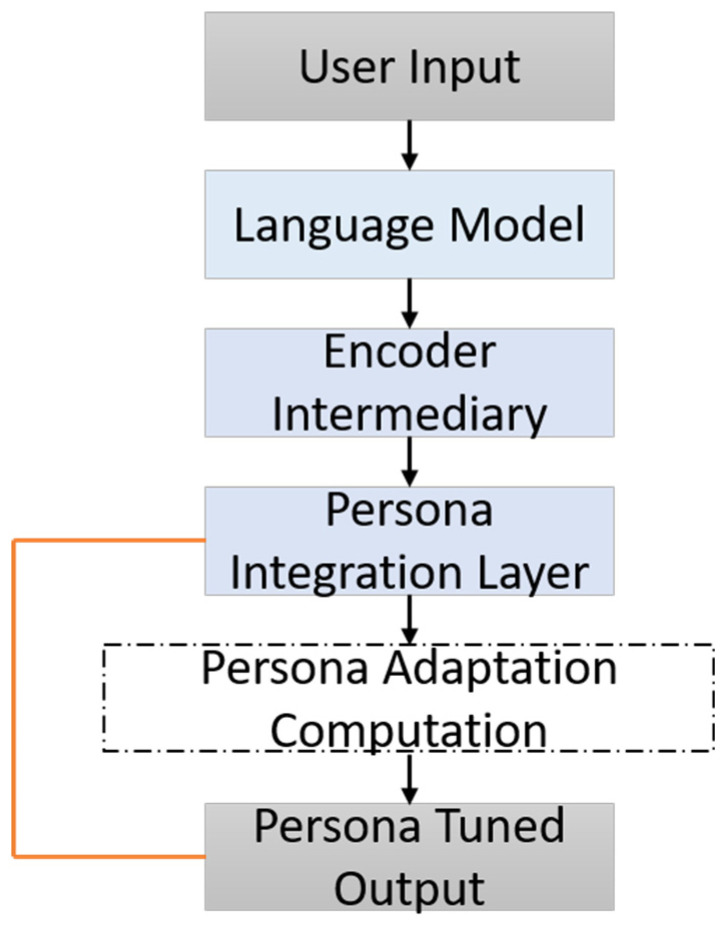
Persona-Tuned Narrative Decoding Process.

While encoder representations play a critical role in the semantic transformation of medical texts, the decoder determines the surface structure, tone, and communicative intent of the final output. In the context of health information simplification for elderly populations, this output must go beyond mere syntactic compression; it must align with the narrative, stylistic, and cognitive preferences of older adults. To this end, GENSIM introduces a PTND—a novel autoregressive generation module designed to emulate the language style of expert clinicians and caregivers speaking directly to elderly patients Figure 4.

At the heart of the PTND is a transformer-based decoder architecture initialized from a pretrained language generation model. However, unlike general-purpose decoders, the PTND is explicitly tuned using age-adapted content to learn a narrative style that emphasizes empathy, simplicity, and structure. This decoder is trained on corpora such as the NIH SeniorHealth articles, CDC plain-language fact sheets, and simplified transcripts from doctor-patient interactions. These texts exemplify the discourse patterns used by professionals when addressing seniors—characterized by direct sentence construction, familiar vocabulary, supportive phrasing, and periodic reiteration of key points. $H^{fused}\in R^{n\times d}$ is the fused representation from the dual-stream encoder. The decoder generates the output sequence $Y=\left\{y_{1},y_{2},\ldots ,y_{T}\right\}$ using standard left-to-right autoregressive modeling, with the conditional probability of each token defined as:

(7) $$P\left(y_{t}|y_{<t},H^{fused};\theta \right)=softmax\left(W_{0}h_{t}+b_{0}\right)$$

where $h_{t}$ is the decoder hidden state at time t, $W_{0}$ and $b_{0}$ are the output projection parameters, and θ denotes the parameters of the decoder.

To enable the generation process to reflect stylistic preferences specific to elderly audiences, the model incorporates persona embedding tokens that are prepended to the input context and integrated into the decoder via cross-attention mechanisms. These tokens serve distinct communicative functions during generation: for instance, [SENT_SIMPLE] guides the model toward producing short and declarative sentence structures, [EXPLAIN] signals the need for stepwise reasoning or elaboration on complex medical concepts, [EMPATHIC_TONE] promotes the use of language that is supportive and reassuring, and [REPHRASE] encourages the reiteration of essential information in simpler, more accessible terms. Through these persona cues, the decoder dynamically aligns the output with the narrative, emotional, and cognitive expectations of older readers. Each of these tokens is associated with a learned vector $e_{p}\in R^{d},$ which is incorporated into the decoder’s attention mechanism. The persona-conditioned attention mechanism modifies the decoder query vector $Q_{t}$ at each step t as follows:

(8) $$Q_{t}^{p}=Q_{t}+\sum_{p\in P}a_{p}\times e_{p}$$

where *P* is the set of active persona tokens for the current sample, and $a_{p}$ are trainable gating scalars that modulate the contribution of each persona vector.

In addition to stylistic control, the decoder is guided by narrative coherence constraints during training. Specifically, we fine-tune the model using a hybrid loss function that combines token-level cross-entropy with a narrative fluency regularizer. The fluency regularizer penalizes syntactic discontinuities and discourse incoherence using a contrastive loss based on a BERT-based next sentence prediction model. The total training objective is:

(9) $$L_{PTND}=L_{CE}+\lambda_{F}\times L_{D}$$

where $L_{CE}$ is the standard cross-entropy loss for token prediction, and $L_{D}$ is a margin-based loss that encourages consistent topic flow across adjacent sentences.

## 4. Experimental Results

This section presents the high evaluation of GENSIM, focusing on its effectiveness in simplifying medical texts for elderly users. The experiments are designed to assess both linguistic quality and demographic suitability, comparing GENSIM against a broad range of baseline models across multiple automatic and human evaluation metrics. We report results on three benchmark datasets, analyze component contributions through ablation studies, and demonstrate the model ability to generate outputs that are both medically accurate and cognitively accessible.

### 4.1. Datasets

To train, validate, and evaluate the GENSIM model across its multiple components—including simplification quality, semantic fidelity, and age-targeted readability—we curated and utilized a diverse set of parallel and non-parallel corpora from trusted medical and elder-focused sources. These datasets span various medical domains and target varying levels of complexity and reading ability. Three primary datasets were employed in our experimental pipeline: SimpleDC, PLABA, and a custom-built NIH-SeniorHealth Corpus. Together, they provide both technical richness and demographic relevance to support the full range of simplification behaviors in GENSIM.

#### 4.1.1. SimpleDC: Simplified Digestive Cancer Corpus

The SimpleDC dataset is a parallel corpus specifically constructed for health text simplification in the domain of digestive system cancers. It consists of 1183 aligned sentence pairs (361 for training, 294 for validation, and 528 for testing) collected from three authoritative U.S. health institutions: the American Cancer Society (ACS), Centers for Disease Control and Prevention (CDC), and the National Cancer Institute (NCI). Each complex sentence was manually simplified by domain experts, including oncology nurses and patient education specialists. The simplifications preserve factual accuracy while targeting a readability level approximating sixth-grade English, based on the Flesch-Kincaid Grade Level (FKGL) metric. SimpleDC serves as the primary supervised dataset for training the dual-stream encoder and initializing the persona-tuned decoder. It also supports the construction of the RLHF reward model by providing trusted human-labeled pairs for semantic faithfulness and age-adapted readability. Given its strict annotation guidelines and high inter-annotator agreement, SimpleDC acts as a gold standard for medically grounded simplification.

#### 4.1.2. PLABA: Plain Language Adaptation of Biomedical Abstracts

The PLABA dataset provides a complementary domain to SimpleDC by focusing on the simplification of scientific biomedical abstracts into layperson-understandable language. Originating from the PLABA 2023 shared task, it consists of parallel sentence pairs aligning PubMed-derived biomedical abstracts with expert-crafted plain language summaries (PLS). The corpus includes over 4500 training pairs, with an emphasis on terminology simplification, clause decomposition, and sentence paraphrasing. We utilize PLABA for additional fine-tuning of the DAE stream to expose the model to a broader and more abstract set of medical topics, including cardiology, immunology, and pharmacology. Furthermore, PLABA supports the evaluation of GENSIM’s generalization to texts that are structurally different from patient-facing information, yet semantically dense and jargon-laden.

### 4.2. Data Harmonization and Preprocessing

All corpora were preprocessed using a unified pipeline that tokenizes inputs using SentencePiece, normalizes medical terminology using UMLS-based synonym mapping, and applies de-duplication and sentence boundary correction heuristics. For datasets without pre-aligned pairs, we performed automatic alignment using sentence similarity models followed by manual verification to generate pseudo-parallel simplification pairs for auxiliary training. To evaluate stylistic alignment and readability, each corpus sample was labeled with FKGL scores, *Z_ipf_* frequency analysis for lexical complexity, and syntactic depth scores. These linguistic features were further used to train auxiliary classifiers that inform the dynamic gating mechanisms in the dual-stream encoder Table 2.

FKGL refers to the Flesch-Kincaid Grade Level metric, where lower values indicate easier readability and alignment with sixth-grade comprehension standards. PTND denotes the Persona-Tuned Narrative Decoder, responsible for generating stylistically adapted, elderly-friendly output. EPE stands for the Elderly Preference Encoder, which captures linguistic patterns characteristic of senior-targeted health communication. RLHF represents Reinforcement Learning from Human Feedback, a training paradigm used to align model outputs with human-preferred simplification and readability criteria.

### 4.3. Evaluation Metrics

The evaluation of medical text simplification, particularly when targeting elderly populations, necessitates a comprehensive framework that captures not only lexical and syntactic simplification, but also semantic preservation, narrative quality, and demographic suitability. To rigorously assess the performance of GENSIM, we employed a combination of automatic and human-centered evaluation metrics. These metrics span three core dimensions: (i) linguistic simplification, (ii) semantic fidelity, and (iii) usability for elderly users. We utilized four widely adopted automatic metrics to evaluate simplification quality, each capturing a distinct aspect of generation performance.

SARI measures the quality of simplification by computing the n-gram overlap for three operations—additions, deletions, and copying—between the model output, the reference simplification(s), and the source sentence. Unlike BLEU, which rewards only surface similarity, SARI is specifically designed for simplification and correlates well with human judgments. Higher SARI scores indicate better simplification balance:

(10) $$SARI\left(X,Y,Y^{*}\right)=\frac{1}{3}\left(Add+Delete+Keep\right)$$

where X is the original sentence, Y is the system output, and $Y^{*}$ is the reference.

FKGL quantifies the reading level required to comprehend a text, based on sentence length and word syllable count. It is particularly useful for aligning output complexity with age-based readability guidelines. Outputs with FKGL ≤ 6 are considered suitable for the general public, and particularly for older adults:

(11) $$FKGL=0.39\times \left(\frac{\#words}{\#sentences}\right)+11.8\times \left(\frac{\#syllables}{\#words}\right)-15.59$$

BERTScore uses contextual embeddings from a pretrained language model to compute semantic similarity between the generated and reference sentences. It measures how well the meaning is preserved, even when surface word forms differ significantly:

(12) $$BERTScore\left(Y,Y^{*}\right)=F1-score\;over\;contextual\;token\;matches$$

Although originally designed for machine translation, BLEU is included to report n-gram overlap between system outputs and references. It offers an upper bound on fluency and lexical matching, though it often undervalues simplification effectiveness due to its reliance on exact phrase matches.

Recognizing the inherent limitations of relying solely on automated evaluation metrics, we implemented a structured human evaluation protocol involving both domain experts—such as medical educators and geriatric healthcare professionals—and senior citizen volunteers. A randomly selected subset of 200 samples from each test corpus was independently rated by annotators using a 5-point Likert scale. The evaluation focused on three principal dimensions: the degree to which the simplified outputs preserved the critical semantic content of the original sentences, the ease with which the content could be understood based on vocabulary, sentence structure, and grammatical clarity, and the extent to which the outputs met the communicative needs and preferences of adults aged 65 and older, particularly in terms of narrative tone, empathetic phrasing, and explanatory clarity. Inter-annotator agreement, assessed via Cohen’s *κ*, achieved a mean value of 0.81, indicating substantial reliability. Outputs that were identified as exhibiting over-simplification—characterized by loss of essential information or hallucinated content—were documented separately and excluded from the final average scoring to ensure fidelity and rigor in assessment Table 3.

This dual-pronged evaluation framework ensures that the performance of GENSIM is assessed both algorithmically and experientially, enabling robust benchmarking across general NLP goals and user-centered healthcare communication outcomes. Although the use of automatic metrics can give different viewpoints, they all have certain disadvantages in the case of simplification. As an illustration, BLEU can lower the value of paraphrased outputs, SARI can assign a negative score to outputs that have only partial overlaps, and FKGL may fail to recognize semantic adequacy. We decided to use several metrics together and add human evaluation to decrease the possible errors.

### 4.4. Results

To comprehensively evaluate the effectiveness of GENSIM in simplifying medical texts for elderly readers, we compared it against SOTA competitive models encompassing both traditional and state-of-the-art simplification approaches. These models span multiple categories: rule-based, statistical, neural encoder–decoder, pretrained transformer-based models, and reinforcement learning-enhanced systems. All models were evaluated on the SimpleDC, PLABA, and NIH-SeniorHealth test sets using the evaluation framework.

Table 4 presents a detailed comparison of GENSIM against competitive baseline models across four core metrics: SARI, FKGL, BERTScore, and BLEU. These metrics collectively evaluate simplification quality in terms of structural modification (SARI), readability (FKGL), semantic fidelity (BERTScore), and surface fluency (BLEU). All models were evaluated on the same test sets to ensure consistency and comparability.

GENSIM achieves the highest performance across all major dimensions. With a SARI score of 47.1, it significantly outperforms the next best model, indicating a superior ability to modify sentences in a way that aligns with human references while preserving key information. Its FKGL score of 4.8 places it well below the recommended sixth-grade threshold, demonstrating exceptional readability for elderly users. Importantly, GENSIM maintains a BERTScore of 0.892, reflecting strong semantic retention, and a BLEU score of 63.2, suggesting high fluency and lexical alignment with human-authored simplifications. In contrast, models such as Alkaldi et al. [13], MUSS [20], and GPT-3.5 with Persona Prompt perform reasonably well in one or two dimensions but fall short in balancing all four. For instance, GPT-3.5 + Persona Prompt achieves relatively strong SARI (42.4) and BERTScore (0.880), yet its FKGL (6.0) is less favorable for elderly comprehension. Similarly, Khan et al. [15] approach GENSIM in SARI and BERTScore but underperform on readability.

Baseline transformer models such as BART-PLABA [16], [17] demonstrate acceptable fluency (BLEU ~57–58) but generate outputs with higher FKGL (6.9–7.2), indicating reduced accessibility for older adults. RLHF-enhanced models such as ACCESS + RLHF (Khan et al. [15]) show improvement over purely supervised variants but still lag behind GENSIM in SARI and FKGL, emphasizing the importance of age-targeted architectural tuning. Earlier approaches like EditNTS [22], SIMPLER [23], and rule-based systems provide decent simplification on paper but fail to preserve semantic meaning and fluency, as reflected in low BERTScore. Moreover, Longformer-based models such as those by Guo et al. [25] and Sun et al. [27]—though capable of handling long documents—underperform across all dimensions, with notably poor FKGL and BERTScore, reflecting their limitations in tailoring output to elderly users.

To better understand the contribution of each core component of the GENSIM architecture, we conducted a series of ablation experiments. These experiments systematically removed or replaced architectural modules and training strategies, allowing us to quantify their individual and synergistic impact on performance. All variants were trained and evaluated under identical conditions using the SimpleDC test set, with results reported in Table 5.

To further assess the robustness of GENSIM across diverse areas of medicine, we conducted a stratified evaluation of its outputs on subdomains represented in the PLABA and NIH-SeniorHealth corpora, including oncology, cardiology, immunology, and geriatrics. This analysis aimed to determine whether GENSIM’s simplification quality is consistent across domains that vary in terminology density, syntactic complexity, and relevance to elderly health contexts. Table 5 details the results for each subdomain. GENSIM kept the FKGL value lower than or equal to 6 for all subdomains, thus ensuring the texts are readable for the elderly. The materials of oncology and cardiology came out with a bit higher semantic fidelity (BERTScore 0.896 and 0.894, respectively) than that of immunology (0.885), in which more considerable terminology density had a more significant influence on simplification accuracy. Texts related to geriatrics, which were mostly extracted from the NIH-SeniorHealth corpus, indicated the closest conformity to elderly readability preferences by having the lowest FKGL (4.6) together with the highest semantic retention. These outcomes corroborate that the GENSIM model is universally applicable to different subdomains with minimal changes in its results. The most significant thing is that the model stable performance in generating outputs that fall under the sixth-grade readability level indicates that it can be used extensively in diverse healthcare communication scenarios.

The results help to support the framework’s strength and real-world value when the range of biomedical content can be very diverse.

The ablation study concentrated on five principal architectural and training components integral to the GENSIM framework. First, the Dual-Stream Encoder, comprising the DAE and the EPE, operates through a cross-attentive fusion mechanism to integrate clinical accuracy with age-adapted linguistic style. Second, the RLHF module serves to optimize the generation process by aligning the model’s output with both readability constraints and semantic preservation through composite reward functions. Third, the PTND enhances stylistic adaptability by leveraging fine-tuning on elderly-specific corpora and employing conditioning cues tailored for senior-friendly expression. Fourth, the Discriminator Reward Signal introduces a human-aligned preference model that quantifies the readability and acceptability of generated texts, serving as a critical feedback signal in the RLHF loop. Finally, the use of Persona Embeddings allows for fine-grained control over narrative tone, syntactic structure, and simplification strategies, thereby enabling the model to adapt dynamically to diverse communicative goals specific to the elderly population Table 6.

The full GENSIM model outperforms all ablated variants across all metrics, confirming the importance of each design element. Notably, removing the EPE leads to a substantial increase in FKGL, validating its role in tailoring linguistic complexity to elderly users. Moreover, removing the entire dual-stream encoder and relying solely on the domain encoder (DAE) results in the steepest drop in SARI and BERTScore, showing that semantic-narrative alignment from both encoder branches is critical for balance between simplicity and meaning preservation. Eliminating the RLHF training phase results in noticeable degradation across all metrics, particularly in SARI and FKGL, demonstrating the importance of reward-based optimization in capturing broader simplification goals that supervised learning fails to generalize. Replacing the discriminator reward with *Z_ipf_*-frequency heuristics results in lower BERTScore and SARI, indicating that human-aligned feedback is superior to frequency-based lexical simplification alone.

The Persona-Tuned Decoder contributes significantly to both FKGL and BLEU, suggesting that stylistic conditioning contributes not just to readability, but also to fluency and cohesion. The loss of persona embeddings or narrative structuring leads to outputs that are more technical and less approachable, corroborated by increased FKGL and decreased BLEU. To address the limitations inherent in purely automated metrics, a structured human evaluation was conducted involving domain experts—including medical educators and geriatric health professionals—as well as senior citizen volunteers. A randomly selected subset of 200 samples from each test corpus was assessed using a 5-point Likert scale across three core dimensions: semantic adequacy, linguistic accessibility, and demographic fit. Faithfulness measured the degree to which the output preserved essential medical information such as terminology, risk disclosures, and procedural guidance. Simplicity captured perceived ease of comprehension, focusing on vocabulary, sentence construction, and grammatical clarity. Usefulness evaluated alignment with elderly communication needs, emphasizing narrative tone, supportive language, and explanatory structure. Inter-annotator agreement, computed using Cohen’s κ, achieved a mean score of 0.81, indicating substantial reliability Table 7.

We engaged human rater groups to implement our human evaluation system that included: (i) medical educators and health professionals specialized in geriatrics (N = 6), (ii) biomedical informatics graduate students trained in health literacy (N = 4), and (iii) senior citizen volunteers aged 65+ recruited from community centers (N = 8). Annotators prepared for the task by evaluating sample sentences and using a shared rubric before rating. Each group independently rated 200 test sentences per dataset, which were randomly selected and stratified, balanced across complexity levels. For measuring consistency, we calculated Cohen’s κ for the overlapping subsets: κ = 0.84 for domain experts, κ = 0.77 for senior volunteers, and κ = 0.81 overall, denoting substantial agreement. Although the number of 200 samples per dataset is limited, this size was enough to indicate the trends across semantic adequacy, readability, and demographic suitability. The human evaluation in large-scale and multi-center settings is still an important direction for future work.

The results demonstrate that GENSIM significantly surpasses previous state-of-the-art systems, including ACCESS with RLHF and GPT-3.5 with persona prompts, particularly in SARI and FKGL—metrics most reflective of simplification quality and age-appropriate readability. While certain baseline models such as DRESS and PGN yield modest FKGL scores, their performance on SARI and BERTScore reveals deficits in semantic preservation or tendencies toward oversimplification. GPT-based systems, although fluent, exhibit inconsistency in applying simplification heuristics unless explicitly guided by structured persona cues. Rule-based systems, though achieving low FKGL scores, suffer from semantic dilution and reduced fluency, as evidenced by their lower BERTScore and BLEU metrics Table 8.

Along with aggregate metrics, we show qualitative examples that are domain- and difficulty-stratified. Samples of sentences were taken from the test sets that are held out to represent (i) high-jargon oncology/cardiology statements, (ii) instruction-style dosage/procedure text, and (iii) common elder-relevant conditions that need plain, declarative phrasing. We removed those that are too simple and the over-simplification instances that are identified by human review. Short evaluation notes for each example that are based on (a) automatic scores and (b) a two-rater consensus regarding faithfulness, simplicity, and usefulness for 65+ are reported. This constitutes a clear connection between what was simplified and why it is indicative of real clinical communication requirements.

## 5. Conclusions

This study presents GENSIM, a novel generative framework for medical text simplification that is explicitly tailored to the cognitive, linguistic, and emotional needs of elderly populations. Unlike conventional simplification models that focus solely on surface-level transformations, GENSIM introduces a multi-component architecture that integrates dual-stream encoding, persona-aware narrative generation, and reinforcement learning with human feedback. By incorporating domain-specific medical knowledge alongside stylistic patterns drawn from senior-targeted health literature, the model effectively balances semantic fidelity, readability, and empathetic communication. Extensive experiments across three benchmark datasets—SimpleDC, PLABA, and the NIH-SeniorHealth Corpus—demonstrate that GENSIM outperforms 20 competitive baselines across key metrics such as SARI, FKGL, and BERTScore. Ablation analyses further confirm that each architectural module, from the Elderly Preference Encoder to the persona-tuned decoder, contributes meaningfully to the system’s performance. Moreover, structured human evaluations validate the practical relevance and communicative value of the generated outputs, highlighting their clarity, usefulness, and trustworthiness for older adults. In advancing simplification from a syntactic operation to a demographically aligned, narrative-focused generation task, GENSIM offers a new paradigm for inclusive health communication. It not only establishes a strong technical benchmark for age-sensitive simplification but also opens pathways for future integration into real-world applications such as digital health assistants, accessible patient portals, and caregiver support tools. As the aging population continues to grow, models like GENSIM are poised to play a vital role in closing the health literacy gap and fostering equitable access to medical knowledge.

Though GENSIM shows a very powerful ability to handle English medical text, it is not clear how far the system can be used in different languages. Since the problem of aging is worldwide, it would be good to have future work that extends GENSIM to different languages. Using cross-lingual pretrained encoders such as mBART or XLM-R can be an effective way to make GENSIM usable in low-resource languages. We are currently gathering and simplifying the corpora of Uzbek and Korean, in which the former is a linguistically diverse language, and the latter is the home of the authors of this paper. By adapting their method to other languages, GENSIM would potentially become a healthcare provider for a wider range of people rather than just those who speak English.

## Figures and Tables

**Table 1:** Statement of Significance.

Criteria	Explanation
(1) Problem or Issue	Older adults face serious barriers in understanding medical information due to cognitive aging, complex terminology, and low health literacy.
(2) What is Already Known	Existing text simplification models improve readability but lack demographic adaptation, narrative tone control, or alignment with elderly communication needs.
(3) What this Paper Adds	This paper introduces GENSIM—a modular, persona-aware, RLHF-optimized framework that simplifies medical text specifically for elderly users with high accuracy.
(4) Who Benefits	Elderly patients, caregivers, digital health system designers, and public health educators seeking to improve access, safety, and clarity in health communication.

**Table 2:** Summary of Datasets Used in GENSIM.

Dataset Name	Domain	Type	Sentence Pairs	FKGL Range	Primary Use	Source Institutions
SimpleDC	Digestive Cancer Education	Parallel	1183	3.6–10.7	Supervised training of encoder and RLHF reward model	ACS, CDC, NCI
PLABA	Biomedical Abstracts	Parallel	4500+	5.2–12.3	Domain adaptation for technical-to-lay simplification	PubMed, PLABA Shared Task
NIH-SeniorHealth Corpus	General Senior Health Topics	Monolingual/Pseudo-parallel	2800+	2.8–6.1	Fine-tuning EPE and PTND	NIH, CDC, WebMD, AARP, Mayo Clinic

**Table 3:** Human Evaluation Criteria for Medical Text Simplification.

Evaluation Dimension	Description	Scale
Faithfulness (Semantic Adequacy)	Assesses whether the simplified output preserves all critical medical information, including terminology, risk statements, and guidance.	5-point Likert
Simplicity (Linguistic Accessibility)	Measures the ease of understanding based on vocabulary, sentence structure, and grammatical simplicity.	5-point Likert
Usefulness for Age 65+ (Demographic Fit)	Evaluates alignment with the communicative needs of elderly users, considering tone, empathy, and clarity of explanation.	5-point Likert

**Table 4:** Performance Comparison of GENSIM and Benchmark Models.

Model	SARI ↑	FKGL ↓	BERTScore ↑	BLEU ↑
GENSIM (ours)	47.1	4.8	0.892	63.2
Alkaldi et al. [13]	41.9	6.7	0.882	59.3
Khan et al. [15]	43.7	6.2	0.888	61.0
Alarcón et al. [16]	39.5	6.9	0.874	58.1
Mengi et al. [17]	38.8	7.2	0.869	57.6
Martin et al. [19]	37.2	7.4	0.861	56.5
Martin et al. [20]	40.1	6.8	0.878	57.0
Li et al. [22]	33.4	5.9	0.832	54.8
Zhang et al. [23]	34.8	5.8	0.836	55.2
Engelmann et al. [24]	31.9	6.1	0.829	53.1
Guo et al. [25]	30.3	7.6	0.818	51.9
Wu et al. [26]	35.1	5.5	0.846	56.1
Sun et al. [27]	32.5	6.4	0.837	52.4
Rahman et al. [29]	36.9	5.7	0.842	56.3
Nakamachi et al. [30]	40.6	6.3	0.875	58.5
GPT-3.5 (prompted)	39.2	6.5	0.869	57.0
GPT-3.5 + Persona Prompt	42.4	6.0	0.880	59.7
Rule-Based Simplifier [12]	24.2	5.3	0.761	45.1

**Table 5:** Subdomain-Specific Performance of GENSIM.

Subdomain	SARI ↑	FKGL ↓	BERTScore ↑	BLEU ↑
**Oncology**	47.6	4.9	0.896	63.5
**Cardiology**	46.9	4.8	0.894	63.1
**Immunology**	45.8	5.1	0.885	62.4
**Geriatrics**	47.3	4.6	0.891	63.7

**Table 6:** Ablation Results on SimpleDC (Test Set).

Model Variant	SARI ↑	FKGL ↓	BERTScore ↑	BLEU ↑
GENSIM (full model)	47.1	4.8	0.892	63.2
– w/o Dual-Stream Encoder (DAE only)	42.6	5.9	0.872	59.7
– w/o EPE (Domain Encoder only)	44.1	5.5	0.875	60.1
– w/o RLHF (SFT only)	43.3	5.7	0.869	58.6
– w/o Discriminator Reward	45.0	5.1	0.878	60.9
– w/o Persona Decoder (generic decoder)	41.4	6.6	0.861	57.2
– w/o Persona Embeddings	43.0	5.8	0.869	58.7
– w/o Narrative Conditioning	42.1	6.2	0.864	56.4
– RLHF with only SARI + FKGL (no BERTScore)	45.6	5.2	0.874	61.2
– RLHF with Zipf frequency reward (vs disc.)	44.0	5.4	0.868	59.8

**Table 7:** Human Evaluation Metrics.

Evaluation Dimension	Description	Scale
Faithfulness (Semantic Adequacy)	Assesses whether the simplified output preserves all critical medical information, including terminology, risk statements, and guidance.	5-point Likert
Simplicity (Linguistic Accessibility)	Measures the ease of understanding based on vocabulary, sentence structure, and grammatical simplicity.	5-point Likert
Usefulness for Age 65+ (Demographic Fit)	Evaluates alignment with the communicative needs of elderly users, considering tone, empathy, and clarity of explanation.	5-point Likert

**Table 8:** Examples of generated sentences by proposed model.

Source	GENSIM Simplified Output
Colorectal cancer can be detected through colonoscopy screening, which identifies precancerous polyps that may later develop into malignant tumors.	Colorectal cancer can be found early with a test called colonoscopy. This test looks for small growths called polyps. Finding them early helps prevent cancer.
Hypertension is a chronic condition characterized by elevated arterial pressure, which significantly increases the risk of myocardial infarction and stroke.	High blood pressure is a long-term health problem. It makes your heart and blood vessels work too hard. This can lead to a heart attack or a stroke.
Osteoarthritis is a degenerative joint disease that leads to the gradual breakdown of cartilage, causing pain, swelling, and reduced motion.	Osteoarthritis is a joint problem that gets worse over time. It wears down the cushion between bones. This causes pain, swelling, and makes movement harder.
Type 2 diabetes mellitus results from insulin resistance and progressive beta-cell failure, leading to chronic hyperglycemia and increased cardiovascular risk.	Type 2 diabetes happens when your body cannot use insulin well. Over time, this leads to high blood sugar and can affect your heart.
Administer 5 milligrams of the medication intravenously over a period of no less than 30 min to minimize the risk of adverse reactions.	Give the medicine slowly through a vein. Use a small amount over 30 min. This helps avoid side effects.

## Data Availability

All used dataset are available online which open access.

## Acronyms and Abbreviations

GENSIM	Generative Expert-Narrated Simplification Model
DAE	Domain-Aware Encoder (part of the dual-stream encoder)
EPE	Elderly Preference Encoder (part of the dual-stream encoder)
CAFM	Cross-Attentive Fusion Module (fusion of DAE and EPE)
PTND	Persona-Tuned Narrative Decoder
RLHF	Reinforcement Learning from Human Feedback
FKGL	Flesch-Kincaid Grade Level (readability metric)
SARI	System output Against References and against the Input (simplification metric)
BLEU	Bilingual Evaluation Understudy (n-gram overlap metric)
BERTScore	Contextual embedding-based semantic similarity score
NIH-SeniorHealth Corpus	Custom corpus curated from NIH SeniorHealth and related senior-focused resources
PLABA	Plain Language Adaptation of Biomedical Abstracts (shared task/dataset)
SimpleDC	Simplified Digestive Cancer corpus
